# Screening, identification, and mechanism analysis of starch-degrading bacteria during curing process in tobacco leaf

**DOI:** 10.3389/fbioe.2024.1332113

**Published:** 2024-03-19

**Authors:** Yan Zhang, Chuandong Jiang, Yangyang Li, Jingguo Sun, Zhenguo Chen, Qiang Zhang, Guangwei Sun

**Affiliations:** ^1^ College of Plant Protection, Shandong Agricultural University, Tai’an, China; ^2^ Hunan Tobacco Research Institute, Changsha, China; ^3^ Hubei Provincial Tobacco Research Institute, Wuhan, China

**Keywords:** tobacco, curing process, starch degradation, functional bacteria, mechanism analysis

## Abstract

Tobacco, a vital economic crop, had its quality post-curing significantly influenced by starch content. Nonetheless, the existing process parameters during curing were inadequate to satisfy the starch degradation requirements. Microorganisms exhibit inherent advantages in starch degradation, offering significant potential in the tobacco curing process. Our study concentrated on the microbial populations on the surface of tobacco leaves and in the rhizosphere soil. A strain capable of starch degradation, designated as BS3, was successfully isolated and identified as *Bacillus subtilis* by phylogenetic tree analysis based on 16SrDNA sequence. The application of BS3 on tobacco significantly enhanced enzyme activity and accelerated starch degradation during the curing process. Furthermore, analyses of the metagenome, transcriptome, and metabolome indicated that the BS3 strain facilitated starch degradation by regulating surface microbiota composition and affecting genes related to starch hydrolyzed protein and key metabolites in tobacco leaves. This study offered new strategies for efficiently improving the quality of tobacco leaves.

## Highlights

•A functional bacterium which can degrade starch efficiently was successfully screened.
•The mechanism of starch degradation by the functional bacterium was successfully analyzed.
•This study provided a novel direction for the extraction of functional bacteria from tobacco leaves and the elucidation of their mechanisms of action. It offered new strategies for efficiently improving the quality of tobacco leaves.

## 1 Introduction

Tobacco is a vital economic crop, and China is the world’s leading producer and consumer of it. Additionally, there is an escalating demand for enhanced tobacco quality, yet a significant disparity persists between the quality of Chinese tobacco and internationally recognized high-quality tobacco. A primary factor contributing to this quality disparity was the retention of substantial amounts of starch and other macromolecules in tobacco leaves post-curing, which significantly compromised the overall quality of the tobacco (Yin et al., 2019; Banožić et al., 2020). High starch content in tobacco leaves, on one hand, renders them stiff, lacking in smooth texture and suppleness, thereby reducing the elasticity of cigarette tobacco. On the other hand, this elevated starch content adversely affects the combustion characteristics, leading to a charred taste and heightened irritation. Consequently, a diminution in both the quality and safety of the cigarettes was observed. Furthermore, it was found that the insufficient degradation of starch led to decreased levels of precursors for aroma compounds, including reducing sugars and amino acids, adversely impacting the aroma and taste of the tobacco leaves. Therefore, the reduction of starch content in tobacco leaves was significantly important for improving their quality and enhancing their usability (Chen et al., 2019; Rong-Hao et al., 2019). Indeed, the curing process was a crucial stage that accentuates the distinctive qualities of tobacco leaves. During this phase, significant degradation of macromolecules like starch occurs, and the synthesis of aroma compounds took place. The facilitation of starch degradation during this stage was identified as a pivotal factor in the improvement of tobacco leaf quality. Despite tireless efforts by researchers, meeting the degradation requirements through the regulation of process parameters has proven challenging (Zuo et al., 2019; Tang et al., 2020).

In recent years, it has been discovered that microorganisms present on the surface of tobacco leaves played a crucial role in enhancing the quality of the curing process. The activities of these microorganisms were indispensable in driving tobacco fermentation, improving tobacco quality, and enhancing aroma. (Yafei et al., 2017; Hu et al., 2021). A series of experiments were conducted to confirm this. For instance, several strains of thermophilic bacteria were isolated from fermented tobacco leaves, and their application to tobacco leaves was found to enhance the aroma of the cured leaves (Gao et al., 2017). A strain of *B. subtilis*, derived from tobacco leaves, was found to improve the fragrance quality of the leaves, increase aroma intensity, and enhance sweetness. Furthermore, increases of 2.52% and 1.92% were observed in the contents of water-soluble total sugars and reducing sugars, respectively (Huang B. et al., 2022a). A significant enhancement of aroma, softness, and flavor in tobacco was achieved through the addition of a co-culture mixture during the curing process. This mixture was comprised of co-cultured *Filobasidium magnum*, importance for fruit flavor, and *Bacillus kochii*, effective in decomposing starch and protein in cured tobacco (Wu et al., 2022). A significant reduction in starch, pectin, and cellulose content was observed in cured tobacco treated with enzyme preparations fermented from *Paenibacillus amylolyticus*. Additionally, a significant increase in total soluble sugars, reducing sugars, and volatile aroma compounds, such as 3-hydroxy-damascone, 2,3-dihydro-3, 5-dihydroxy-6-methyl-4 H-Pyran-4-one, ethyl palmitate, and ethyl linolenic acid, was noted (Gong et al., 2023). However, numerous challenges were encountered in the microbial degradation of starch in tobacco leaves, such as low technological maturity and limited application scale, hindering the identification of efficient strains for tobacco starch degradation. And in the tobacco curing process, the high temperatures are unsuitable for the growth and degradation activities of most microbes. Additionally, the presence of metabolites like nicotine, anabasine, and nicotinic acid in tobacco significantly inhibits the growth and degradation activity of microbes. Additionally, there have been few reports on the exploration of microorganisms involved in starch degradation during the curing process of tobacco leaves, attributed to the higher temperatures. The identification and investigation of functional bacteria capable of degrading starch during the curing process were especially crucial.

In this study, functional bacterial strains capable of starch degradation were successfully screened for the first time from the rhizosphere soil and the surface of tobacco leaves. These strains were identified as *B. subtilis* through 16S rRNA amplification and phylogenetic analysis. Furthermore, the application of the strain to the surface of tobacco leaves was found to effectively promote the degradation of macromolecules, including starch. Additionally, the mechanism underlying starch degradation by this functional bacterium was elucidated through the integration of metagenomics, transcriptomics, and metabolomics analyses. This research has provided new directions for the extraction of highly efficient functional bacteria capable of degrading starch during the curing process and for elucidating their mechanisms of action. New strategies for enhancing the quality of tobacco leaves during the curing process were also presented.

## 2 Methods and materials

### 2.1 Materials and locations

The flue-cured tobacco variety used was Yunyan 87, with seeds provided by the Hubei Tobacco Science Institute. Planting and functional microbial screening of the rhizosphere soil were conducted at the Baiyangba Tobacco Station experimental site in Lichuan City, Enshi Prefecture, Hubei Province (108°6709′E, 30°3308′N). Fresh leaves from the middle part of moderately mature tobacco plants were selected for experimental analysis. Standard harvesting and curing methods were followed to ensure uniform distribution of the leaves. The curing process was carried out in the curing barn at Baiyangba Tobacco Station in Enshi City.

### 2.2 Strain collection and isolation screening

10 g of tobacco root soil were collected and placed in a sterilized triangular bottle, to which 90 mL of sterile water was added. In a separate procedure, 10 g of tobacco leaves were finely chopped using sterilized scissors and then immersed in 90 mL of sterilized distilled water. The mixture was incubated on a shaker set at 37°C and 230 rpm for 4 h, resulting in the formation of the original solution. Microbial strains were isolated and screened using a stepwise dilution method. Ten different dilution gradients, ranging from 1–10^–9^, were prepared. Each dilution was then plated onto solid Luria-Bertain (LB) medium and incubated at 37°C for 24 h. Individual colonies were selected and purified using streaking techniques on LB solid medium to obtain pure isolates. The purified strains were preserved by adding 15% sterile glycerol and storing them at −80°C. Holes were made in starch or protein agar medium, and 10 uL of the activated fermented broth from the bacterial cultures, after 24 h of growth, was dropped into each hole of the agar medium. The plates were then inverted and placed in a 37°C incubator for 24 h. After incubation, iodine solution was added to observe the formation of transparent zones around the holes.

The activated bacterial cell suspensions were diluted and spread onto solid starch agar plates medium. After allowing the liquid to dry, the plates were inverted and placed in a 37°C incubator for 1 day of incubation. When the bacterial colonies attained a specific circular size, a predetermined amount of iodine standard solution was applied at the edge of each colony. After sufficient penetration, the diameter of the transparent zone (H) and the diameter of the colony (C) were measured using a caliper. The ratio of H to C (H/C) was calculated. The bacterial strain with a larger hydrolysis zone was selected for further determination of starch hydrolysis zone size and measurement of amylase and protease activity.

### 2.3 Genome amplification and sequencing

#### 2.3.1 16S rRNA gene extraction and amplification

The universal bacterial 16S rRNA primers 27F and 1492R were utilized for PCR amplification. (Coenye et al., 1999). The 16S rRNA primer sequences are as follows: 27F Forward: 5′-AGA​GTT​TGA​TCC​TGG​CTC​AG-3′, 1492R Reverse: 5′-GGT​TAC​CTT​GTT​ACG​ACT​T-3'. PCR amplification conditions included: pre-denaturation at 94°C for 5 min, denaturation at 94°C for 1 min, annealing at 50°C for 1 min, extension at 72°C for 3 min and 15 s, over 30 cycles, and final extension at 72°C for 10 min. PCR amplification was performed in a 25 μL reaction system comprising 1.5 μL of each primer, 1.5 μL of template DNA, 10 μL of ddH_2_O, with the total volume made up to 25 μL. Amplification was conducted using a mvevcler@PCR instrument from Bio-Rad, United States. Post-amplification, 5 μL of the PCR product was loaded onto a 2% agarose gel for electrophoresis at 5V/cm. The results were visualized with a Bio-Rad gel imaging system. PCR products with satisfactory amplification were recovered and forwarded to Wuqing Biotech Co., Ltd. for sequencing. The GenBank accession number(s) of nucleotide sequence(s) was SUB14037676 BS-3 OR939273.

#### 2.3.2 Homology analysis and construction of a phylogenetic tree

The obtained genes were sequenced, and a comparative analysis of homology was conducted on NCBI. The comparison results were used to construct a phylogenetic tree using the MEGA4.0 system (Kumar et al., 2004).

### 2.4 Aerosol application on tobacco leaves and sample determination

Mixing the strain fermentation broth and distilled water in a 1:1 ratio, and uniformly spraying it on both sides of the tobacco leaves until they are evenly coated but not dripping.

#### 2.4.1 The determination of physiological parameters in roasted tobacco leaves

Tobacco leaves sprayed with water and functional microbial fermentation broth are to be placed in a drying chamber for dense curing (three-stage curing). The curing chamber is set to temperatures of 38°C, 40°C, 42°C, 44°C, and 46°C, respectively. Samples from the tobacco leaves will be collected at each temperature to determine enzyme activities, including peroxidase (POD), superoxide dismutase (SOD), polyphenol oxidase (PPO), various amylases (α-amylase, β-amylase, and total amylase), and starch branching enzyme (SBE), along with the measurement of malondialdehyde (MDA) and starch content. SOD was determined using the nitroblue tetrazolium (NBT) spectrophotometric method (Zhou and Prognon, 2006). Additionally, the guaiacol method was used for determining POD (Doerge et al., 1997). PPO was determined using the p-phenylphenol spectrophotometric method (Houlong et al., 2017). Amylases were determined using the 3,5-dinitrosalicylic acid (DNS) spectrophotometric method (Keharom et al., 2016). SBE was determined by spectrophotometer (Jiang et al., 2003) MDA was determined by the thiobarbituric acid (TBA) reaction (Davey et al., 2005).

#### 2.4.2 The extraction and determination of samples for metagenomic, transcriptomic, and metabolomic analysis

Tobacco leaves treated with the functional bacteria were placed in a curing barn for intensive curing. Upon the curing barn temperature reaching 40°C, leaf samples were collected and immediately transported to the laboratory on ice, followed by storage in a −80°C freezer for preservation. Subsequent omics analysis was conducted at Beijing Novogene Co., Ltd. The samples of metagenome were analyzed at Illumina Novaseq6000 (Illumina Corporation, Illumina, San Diego, CA, United States). The samples of transcriptome were analyzed at Illumina NovaSeq6000 platform (Illumina Inc., San Diego, CA; Novogene Co., Ltd.; Beijing, China). The samples of metabolome were analyzed at Q Exactive™ HF-X (Thermo Fisher, Germany), Vanquish UHPLC (Thermo Fisher, Germany) and Hypesil Gold column (100 × 2.1 mm, 1.9 μm) (Thermo Fisher, United States). Good QC reproducibility was exhibited by the experimental process, and stability was maintained in the analysis system. For data analysis, multivariate statistical analysis methods were employed using the SIMCA-P11 software.

#### 2.4.3 Metagenomics-related analysis

In this study, the richness and diversity of bacterial communities among different treatments were evaluated and compared using OTUs, Chao1, Simpson, and Shannon indices (Nossa et al., 2010). The microbial richness in differently treated tobacco leaves was assessed by analyzing their OTUs, Chao1, and Shannon indices. The coverage of OTU species in the leaf samples was also calculated.

#### 2.4.4 Transcriptomic analysis

The differential expression of genes was analyzed using DESeq2 (Love et al., 2014), with criteria based on variable importance in the projection >1.0 (Chong & Jun, 2005; Farrés et al., 2015), FC > 1.5, *p* < 0.05. The annotation and visualization of KEGG and GO terms were performed using the ggplot2 software (Huerta-Cepas et al., 2017). Enrichment analysis was conducted using a clustering analysis tool, and the data were visualized using R programming language and TBtools for visualization (Chen et al., 2020). R (Version 3.5.0) WGCNA package was used to process the output gene co-expression network to screen out the core genes and perform visualization analysis (Diboun et al., 2006). The CAZymes was annotated by dbCAN (https://bcb.unl.edu/dbCAN2/) (Yin et al., 2012).The Submission ID of RNA-seq was SUB14056576.

#### 2.4.5 Screening of differentially expressed metabolites and analysis of metabolic pathways

Orthogonal partial least squares discriminant analysis (OPLS-DA) was utilized for the analysis of differentially expressed metabolites (DEMs), and a corresponding PLS-DA model was established. The R2 and Q2 values of the random model were obtained. Important metabolites were selected based on the PLS-DA model results, using VIP values with a threshold of VIP > 1.0 and *p* < 0.05 for DEM screening (Usadel et al., 2009). The numbers of upregulated and downregulated DEMs were calculated, and the expression patterns of the top ten DEMs, based on VIP values, were ranked. For further analysis of DEM differences, identified metabolites were functionally and categorically annotated in the Human Metabolome Database (HMDB), and a clustering heatmap was generated to represent the differential expression patterns of various metabolites.

### 2.5 Data analysis

Statistical and analytical processing of all data in this study was conducted using software such as DPS data processing system, SPSS Statistics, Origin 9.0, R studio, and Excel 2010.

## 3 Results

### 3.1 Selection of functional bacteria

#### 3.1.1 Selection of starch-degrading functional bacteria

**FIGURE 1:**
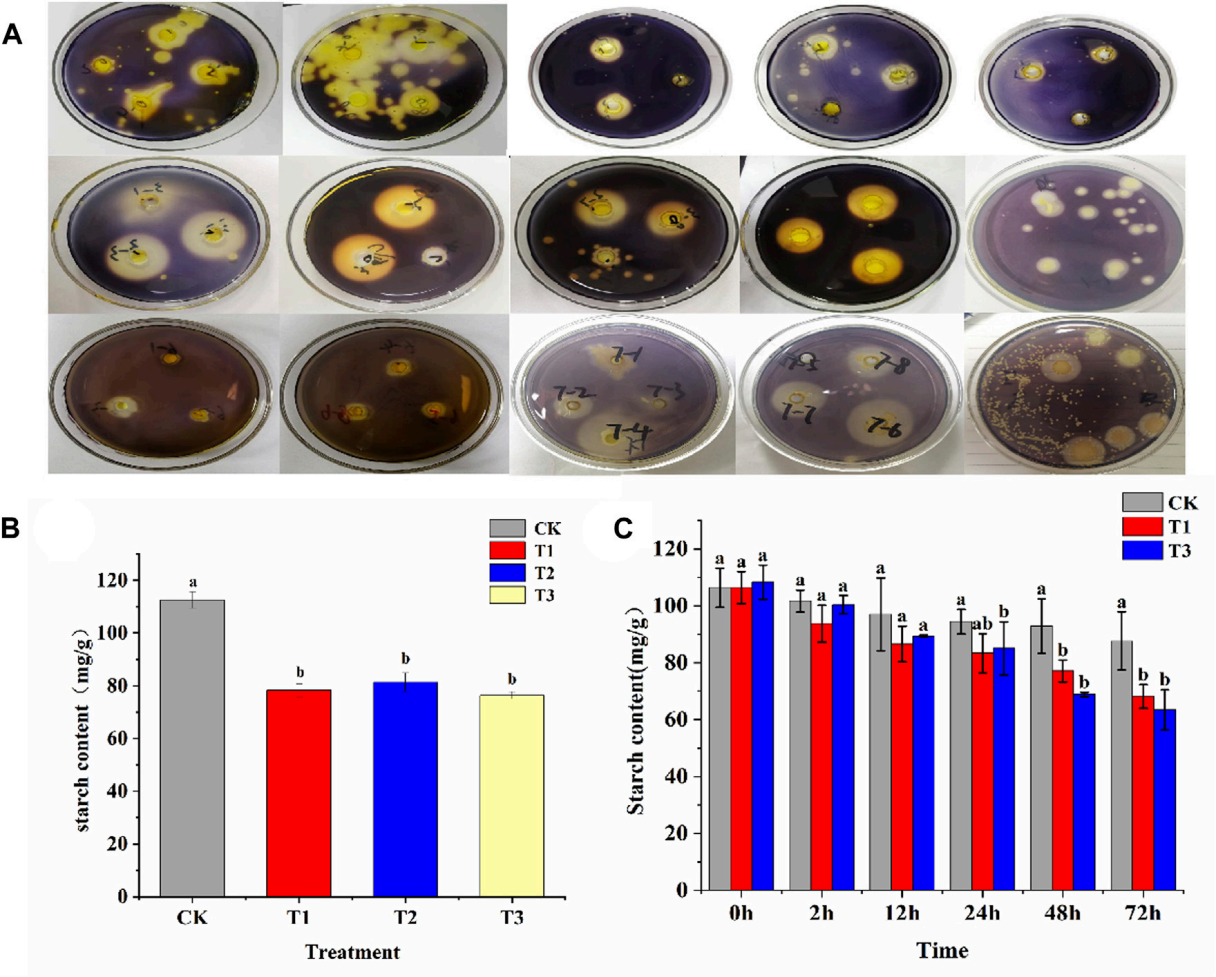
Screening of functional bacteria and their effect on starch content. **(A)** Preliminary screening of functional bacteria for starch degradation in tobacco leaves was conducted based on selective culture media; **(B)** At 24 h, the starch degradation in fresh tobacco leaves by T1, T2, and T3 strains was observed. **(C)** At 2, 12, 24, 48, and 72 h, the starch degradation in fresh tobacco leaves by T1 and T3 strains was observed.

A total of 63 bacterial strains and 3 fungal strains were isolated from tobacco rhizosphere soil and tobacco leaf surfaces by dilution plating method. Among these, 36 strains were capable of forming transparent zones on starch-selective culture medium (Figure 1A). The 19 strains, exhibiting larger hydrolysis zones, were selected for subsequent steps. Enzyme activity was measured after 24 h of liquid culture, revealing that 14 strains exhibited an H/C ratio greater than 1.5. Of these, strains 12, 13, and 14 showed the highest amylase activity, with values of 150.59, 116.84 U/mL, and 126.96 U/mL, respectively (Supplementary Table S1). Three bacterial strains were selected for fermentation broth preparation and designated as T1, T2, and T3. These strains were uniformly sprayed onto the surfaces of tobacco leaves, accompanied by an equal amount of sterile water serving as the CK. After 24 h, the starch content in the tobacco leaves was measured. It was observed that the starch content in all three treatment groups was significantly lower compared to the CK group, indicating the pronounced effect of these treatments on starch degradation in tobacco leaves. Additionally, lower starch content was exhibited in the T1 and T3 treatments compared to the T2 treatment, suggesting a more effective starch degradation in tobacco leaves by the T1 and T3 strains (Figure 1B). Subsequently, T1, T3, and an equal amount of sterile water were uniformly sprayed on both sides of the tobacco leaves. The starch content of various treatments at different times was measured. It was found that no significant difference in starch content existed between the treatment and control groups during the first 12 h. After 24 h of treatment, a decrease in starch content was observed in T1, though not significantly, while a significant decrease (*p* < 0.05) was noted in T3. Post 48 h, significant reductions in starch content were seen in both T1 and T3 compared to the CK group. Additionally, T3 exhibited a slightly lower starch content than T2 (Figure 1C). The degradation rates of CK, T1 and T3 were compared. The results indicated that the degradation rates of T3 were higher than those of CK and T1 within 72 h. Additionally, a rapid increase in T3’s degradation rate was observed, rising from 24.56% to 33.47% between 24 and 48 h, signifying its superior performance during this period (Supplementary Table S2). Therefore, T3 strain was selected for further experiments.

#### 3.1.2 Identification of functional bacteria

The amplified 16S rDNA products from the strain were purified, sequenced, and subjected to comparative analysis with nucleotide sequences in Genebank to identify the functional bacteria T3. It was indicated that strain T3 exhibited high homology with *B. subtilis* (MN945444). A phylogenetic tree including strain T3 and closely related strains was constructed using the neighbor-joining method (Supplementary Figure S1), revealing that strain T3 had the closest genetic affinity with *B. subtilis* MF957285. Consequently, strain T3 was identified as *B. subtilis* and designated as BS3.

### 3.2 Effects of BS3 strain on physiological indicators of tobacco leaves during curing process

**FIGURE 2:**
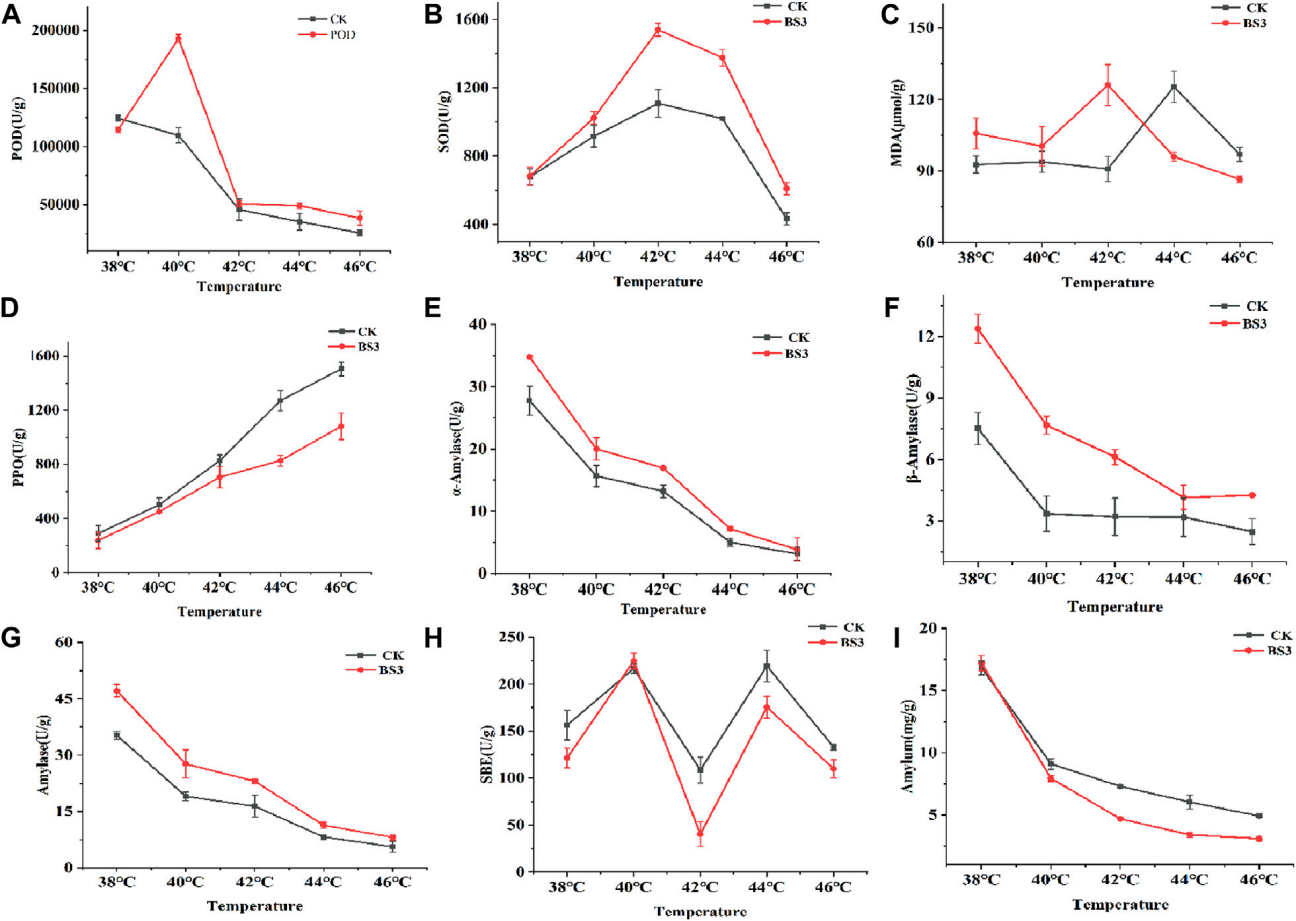
Changes in physiological indicators of tobacco leaves in different treatment groups during curing: **(A)** SOD activity; **(B)** POD activity; **(C)** MDA content; **(D)** PPO activity; **(E)** α-amylase activity; **(F)** β-amylase activity; **(G)** total amylase activity; **(H)** SBE activity; **(I)** Starch content. *RT: Cool to room temperature after curing.

Enzyme activity were crucial factors that influence the degradation of starch of tobacco leaf during curing process (Lacerda et al., 2018; Loh et al., 2018). Consequently, the activities of enzymes related to starch degradation (SOD, POD, PPO, α-amylase, β-amylase, total amylase, SBE) at temperatures of 38°C, 40°C, 42°C, 44°C, and 46°C were measured during the curing process (Figures 2A, B, D–H). The results indicated that the addition of BS3 significantly increased the activities of SOD and POD enzymes, thereby facilitating the degradation and conversion of tobacco leaf constituents. Concurrently, a gradual decline in PPO activity was observed, signifying a reduced susceptibility to browning reactions. Furthermore, significant increases in the activities of α-amylase, β-amylase, total amylase, and SBE were observed, highlighting BS3’s effective enhancement of starch degradation in tobacco leaves. Subsequently, substances related to the tobacco leaf curing process were measured (Figure 2C). The results showed that an earlier increase in MDA content occurred compared to the CK group, reaching a high level before 42°C, which contributed to the acceleration of the tobacco curing process and enhanced the leaf’s resistance to environmental stress. Tobacco leaf samples were collected at 38°C, 40°C, 42°C, 44°C, 46°C, and at the end of curing when cooled to room temperature. Subsequently, all the samples were crushed, and their starch content was determined (Figure 2I). The results showed a decrease in the total starch content of the tobacco leaves over time. It was observed that the starch content in both the CK and BS3 treatments significantly declined from 38°C to 40°C. The degradation rates in the CK and BS3 treatments were 45.91% and 57.01%, respectively. Notably, the starch degradation rate in the BS3 treatment group was consistently higher than that in the CK group thereafter. At the end of the curing process, the starch degradation rate in the BS3 group was found to be 13.8% higher than that in the CK group. These results indicated that BS3 is capable of enhancing crucial enzyme activity and related substances in tobacco leaves, thus achieving efficient starch degradation and accelerating the curing process.

### 3.3 Effects of applying functional bacteria on the diversity analysis of bacteria and fungi in tobacco leaves

**FIGURE 3:**
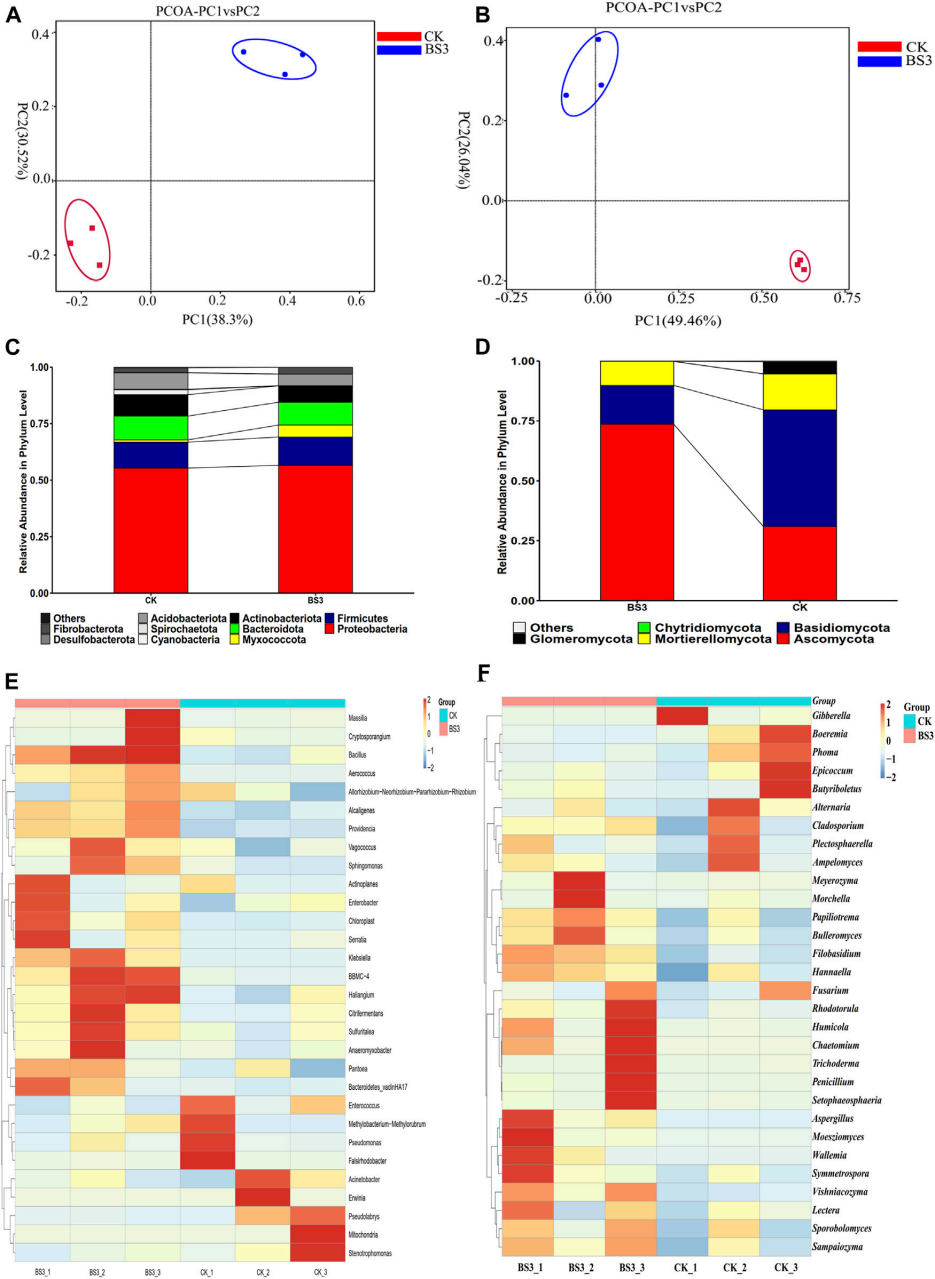
Analysis of metagenomic under different treatments. **(A)** the principal coordinate analysis (PCoA) of bacterial communities in tobacco leaves; **(B)** the PCoA of fungal communities in tobacco leaves; **(C)** the relative abundance of bacterial phyla in tobacco leaf samples; **(D)** the relative abundance of fungal phyla in tobacco leaf samples; **(E)** the hierarchical clustering analysis of dominant bacterial genera; and **(F)** the hierarchical clustering analysis of dominant fungal genera.

Bacterial community richness was analysed by OTUs, Chao1, Shannon and Simpson index (Supplementary Table S3). The results indicated that the OTUs, Chao1, and Shannon indices of tobacco leaf samples treated with the BS3 strain were higher than those in the CK group, with the Shannon index in the BS3 treatment group being significantly higher than that in the CK group (*p* < 0.05). Additionally, a significantly lower Simpson index was observed compared to that of the CK group (*p* < 0.05). These findings indicate an increase in species diversity and community abundance in tobacco leaves in the BS3 group compared to the CK group. Moreover, the OTU species coverage exceeded 0.99, indicative of high library coverage, which more truthfully and objectively reflects the bacterial species diversity. Furthermore, PCoA analysis revealed a clear separation in the distribution of sample points between the CK and BS3 treatment groups, indicating a significant effect of BS3 treatment on the bacterial community structure of tobacco leaves (Figure 3A). At the phylum level, *Proteobacteria* (over 55%) were identified as the dominant phyla in both the CK and BS3 groups. Furthermore, the abundance of *Myxococcota* in the BS3 group was higher than in the CK group, while the abundance of *Actinobacteria* in the BS3 group was lower than in the CK group (Figure 3C).

Hierarchical clustering analysis was performed on the heatmap of the top 30 genera abundance (Figure 3E). Differences in bacterial community structure between the CK and BS3 groups were observed. A remarkable increase in the abundance of genera such as *Massilia, Bacillus, Cryptosporangium, Klebsiella, Haloferula, Citrobacter, Sulfuritalea, Alcaligenes,* and *Anaeromyxobacter* was noted, while a significant reduction in genera such as *Stenotrophomonas, Pseudomonas, Erwinia, Acinetobacter, Falsirhodobacter,* and *Enterococcus* was observed in the BS3 group compared to the CK group. The bacteria of *Bacillus* can secrete various amylases, which can degrade starch and produce organic acids (Ma et al., 2023). Moreover, it has been found that certain anaerobic bacteria within the Anaeromyxobacter genus can degrade starch into organic acids and gases, such as acetic acid, propionic acid, butyric acid, hydrogen, and carbon dioxide (Heng et al., 2022). The production of these organic acids and gases was found to further promote starch degradation. These results indicated that the application of the BS3 strain influenced the bacterial community structure of tobacco leaves to a certain degree. Additionally, the application of BS3 significantly enriched bacteria known to facilitate starch degradation, thereby accelerating the process.

Regarding fungal diversity, the richness and diversity of fungal communities in tobacco leaves were analyzed. An OTU species coverage greater than 0.99 was observed, indicating high library coverage and thus providing a more accurate reflection of fungal species diversity. In addition, the OTUs and Chao1 of fungal communities in tobacco leaves treated with the BS3 strain were found to be significantly lower than those in the CK group (*p* < 0.05), whereas the Shannon index was higher, albeit not significantly different from the CK group. It was indicated that the BS3 strain reduced the abundance of fungal communities while increasing the diversity of fungal species, suggesting a significant role of the BS3 strain in altering the fungal diversity in tobacco leaves (Supplementary Table S4). PCoA analysis was performed, revealing significant separation between the sample points of the CK and BS3 treatment groups, indicating BS3’s significant effect on the fungal community structure in tobacco leaves (Figure 3B). At the phylum level, Ascomycota and Basidiomycota were the dominant phyla in the BS3 and CK groups, respectively. Furthermore, the relative abundances of *Zygomycota* and *Glomeromycota* in the BS3 group were lower compared to those in the CK group (Figure 3D).

Hierarchical clustering analysis of the top 30 genera was conducted using a heatmap to further analyse the compositions of fungal community structures (Figure 3F). Variations in fungal community structures among the different treatments were observed in the heatmap analysis. In the BS3 group, a significant increase in the relative abundance of several genera was observed, including *Rhodotorula, Humicola, Chaetomium, Trichoderma, Penicillium, Setophaeosphaeria, Aspergillus, Moesziomyces, Wallemia, Morchella, Bulleromyces,* and *Sporobolomyces*. Furthermore, a significant reduction was observed in the relative abundance of several other genera, including Gibberella, Phoma, Epicoccum, Butyriboletus, Alternaria, and Cladosporium. Among these, the genera Trichoderma, Aspergillus, and Penicillium were noted for secreting multiple amylases and cellulases to degrade starch and cellulose into various sugars (Matsuzawa et al., 2021; Zhang et al., 2021). These results suggested that the BS3 strain significantly enriched the fungal communities that can degrade starch, which could accelerate starch degradation in tobacco leaves.

### 3.4 Transcriptome analysis of tobacco leaves after the application of BS3

**FIGURE 4:**
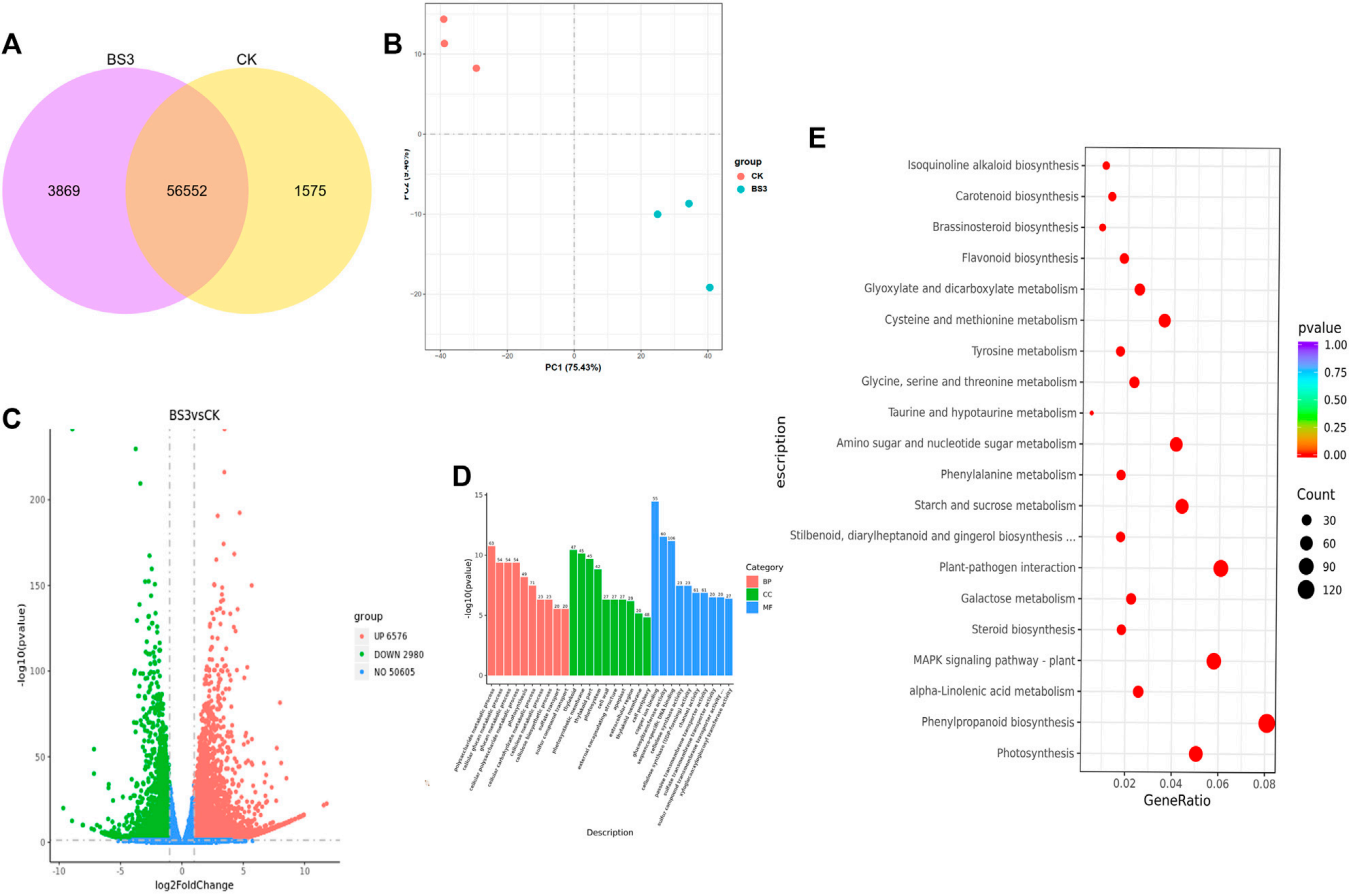
Analysis of gene expression levels in the transcriptome of tobacco leaves. **(A)** Co-expression Venn diagram; **(B)** Principal component analysis (PCA) plot; **(C)** Volcano plot of differentially expressed genes (DEGs); **(D)** GO functional analysis of DEGs in different treatments; red represented molecular functions, green represented biological process functions, blue cellular components; **(E)** KEGG enrichment analysis of DEGs.

To further investigate the mechanisms by which the BS3 strain accelerates starch degradation in tobacco leaves, RNA-seq analysis of both the BS3 and CK groups was conducted. Principal Component Analysis (PCA) revealed distinct clustering and separation between these groups, indicating significant differences in gene transcription levels (Figure 4B). Transcriptome analysis indicated 60,421 and 58,127 expressed genes in the BS3 and CK groups, respectively, with 56,552 genes co-expressed and 3,869 and 1,575 genes specifically expressed in the BS3 and CK treatments, respectively (Figure 4A). Comparative transcriptome data revealed 10,556 differentially expressed genes (DEGs) in the BS3 group versus the CK group, applying a threshold (VIP>1.0, FC > 1.5, *p* < 0.05), including 6,576 upregulated and 2,980 downregulated genes (Figure 4C). These results suggest that multiple physiological processes might be enhanced by the influence of functional bacteria.

Gene Ontology (GO) enrichment analysis was performed to investigate the functions of the 10,556 DEGs (Figure 4D). This analysis revealed that the DEGs were significantly classified into three categories: molecular functions, biological process functions, and cellular components. In the molecular functions category, the most abundant genes corresponded to copper ion binding, glucosyltransferase activity, and sequence-specific DNA binding. In the biological process functions category, the most abundant processes were polysaccharide metabolic processes, cellulosic and cellulose metabolic processes, highlighting the enrichment of DEGs in the polysaccharide metabolism pathway. In the cellular components category, genes associated with organelles such as plastids, thylakoid membranes, and photosystems were most abundant, suggesting that the differentially expressed genes are primarily involved in metabolic processes related to photosynthesis. This indicates that under the influence of BS3, genes related to carbohydrate metabolism were significantly enriched, potentially accelerating starch degradation.

Kyoto Encyclopedia of Genes and Genomes (KEGG) pathway enrichment analysis was performed (Figure 4E), identifying the metabolic pathways involving DEGs. The analysis revealed that DEGs were primarily enriched in 115 pathways, with 33 pathways showing significant differences. The top 20 pathways of greatest statistical significance, based on the *p*-value, were selected for a KEGG enrichment scatter plot. The results indicated significant enrichment of the phenylpropanoid biosynthesis signaling pathway, starch and sucrose metabolism, phenylalanine metabolism, and the metabolism of glycine, serine, threonine, and cysteine with the application of BS3. DEGs related to secondary metabolism signaling, sugar substance metabolism, and amino acid metabolism may influence starch degradation.

### 3.5 Construction of weight gene co-expression network

**FIGURE 5:**
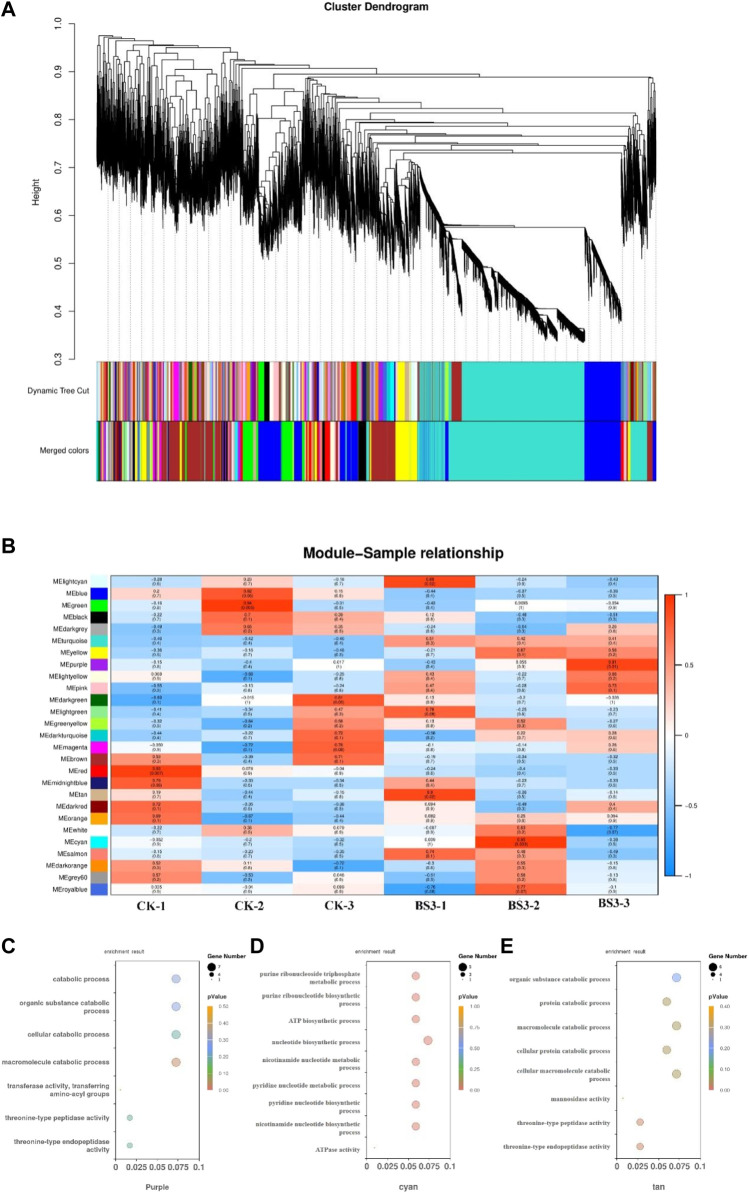
Construction of weighted gene co-expression network **(A)** Clustering dendrogram of genes and module division. **(B)** Module-tissue association in BS3. **(C)** Purple module GO analysis result. **(D)** cyan module GO analysis result. **(E)** tan module GO analysis result.

All 38,682 genes were retained for WGCNA unsigned co-expression network analysis. The BS3 weight gene co-expression network was successfully constructed by WGCNA method, and the network was divided into 27 modules (Figures 5A, B). The three modules purple, tan and cytan were significantly positively correlated with BS3. A total of 517 genes were encompassed by the purple module, 462 genes by the tan module, and 344 genes by the cyan module. The purple and tan (0.91 and 0.90) module, which is positively related to macromolecular organic compounds. Modules positively associated with purine and pyrimidine metabolism include cyan (0.95) modules. Tissue-specific modules may be involved in tissue-specific biological processes. In order to verify the reliability of co-expression network construction and tissue-specific module, the tissue-specific module of starch degradation in tobacco by BS was analyzed.

Analysis of the three tissue-specific modules of BS revealed that these modules are significantly enriched in the biological processes of organic substance degradation and pyrimidine and purine metabolic pathways. Further GO enrichment analysis was conducted on these modules (Figures 5C–E). The results showed significant enrichment of organic macromolecule decomposition metabolic processes and pyrimidine and purine metabolic processes in these modules. The purple and tan modules were notably enriched in organic macromolecule decomposition metabolic processes such as the organic substance catabolic process (GO:1901575), macromolecule catabolic process (GO:0009057), and cellular macromolecule catabolic process (GO:0044265). The Cyan module was rich in pyrimidine and purine metabolic processes, as well as ATP metabolic processes, such as nucleotide biosynthetic process (GO:0009165), pyridine nucleotide metabolic process (GO:0019362), and ATP biosynthetic process (GO:0006754).

### 3.6 Metabolomics analysis of tobacco leaves after the application of BS3

**FIGURE 6:**
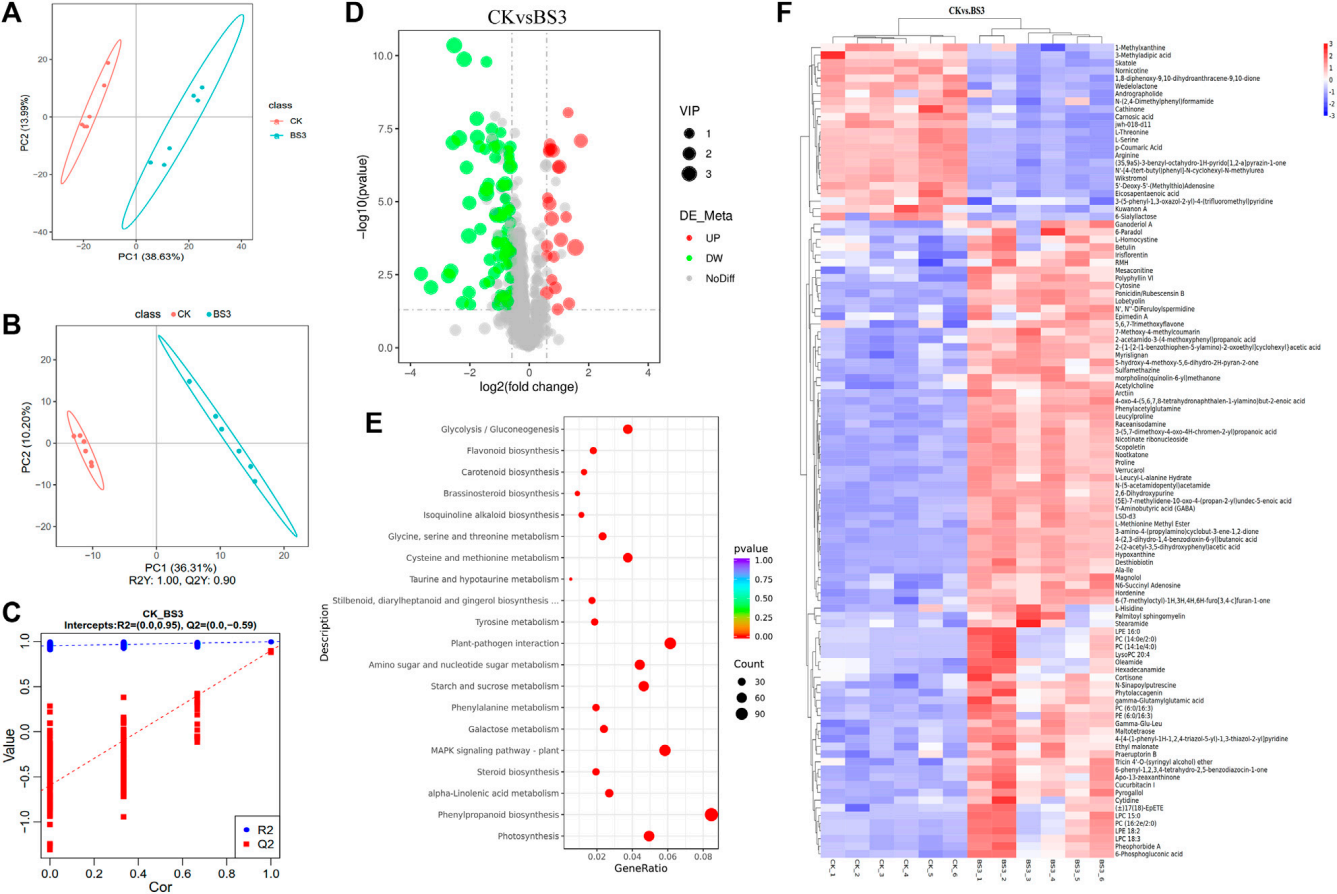
Analysis of the metabolomics tobacco leaves. **(A)** Gene expression level analysis; **(B,C)** Partial least squares-discriminant analysis (PLS-DA) plot; **(D)** Volcano plot of differentially expressed metabolites (DEMs); **(E)** KEGG enrichment analysis of DEMs; **(F)** Cluster analysis of DEMs.

A metabolomic analysis of the two treatment groups was conducted to further explore the mechanisms behind BS3’s effects on starch degradation in tobacco leaves. A total of 106 metabolites exhibiting significant disparities were identified, which included 82 upregulated metabolites and 24 downregulated metabolites following the application of BS3 (Figure 6D). The PCA results demonstrated a clear divergence in the clustering and segregation of the BS3 and CK groups (Figure 6A). The outcomes of the OPLS-DA model (*p* < 0.05, VIP>1) aligned with those derived from the PCA analysis (Figures 6B, C), suggesting that BS3 might influence multiple physiological processes.

Metabolites were annotated and classified using the MetabolomeData_x0002_base (HMDB) and presented as a heatmap (Figure 6F). The results indicated that upregulated differentially expressed metabolites (DEMs) included glycosidic metabolites such as scopolin and nicotinamide ribonucleotide, and organic acid metabolites like glucose-6-phosphate and fumaric acid following the application of BS3. Conversely, amino acid metabolites such as nornicotinoid, arginine, serine, and threonine were identified as downregulated DEGs. Subsequent KEGG pathway enrichment analysis on the DEMs (Figure 6E) identified the top three enriched pathways: cysteine and methionine metabolism, porphyrin and chlorophyll metabolism, and monoterpenoid biosynthesis, indicating that these metabolites are related to amino acids and alkaloids.

### 3.7 Multi-omics association analysis

**FIGURE 7:**
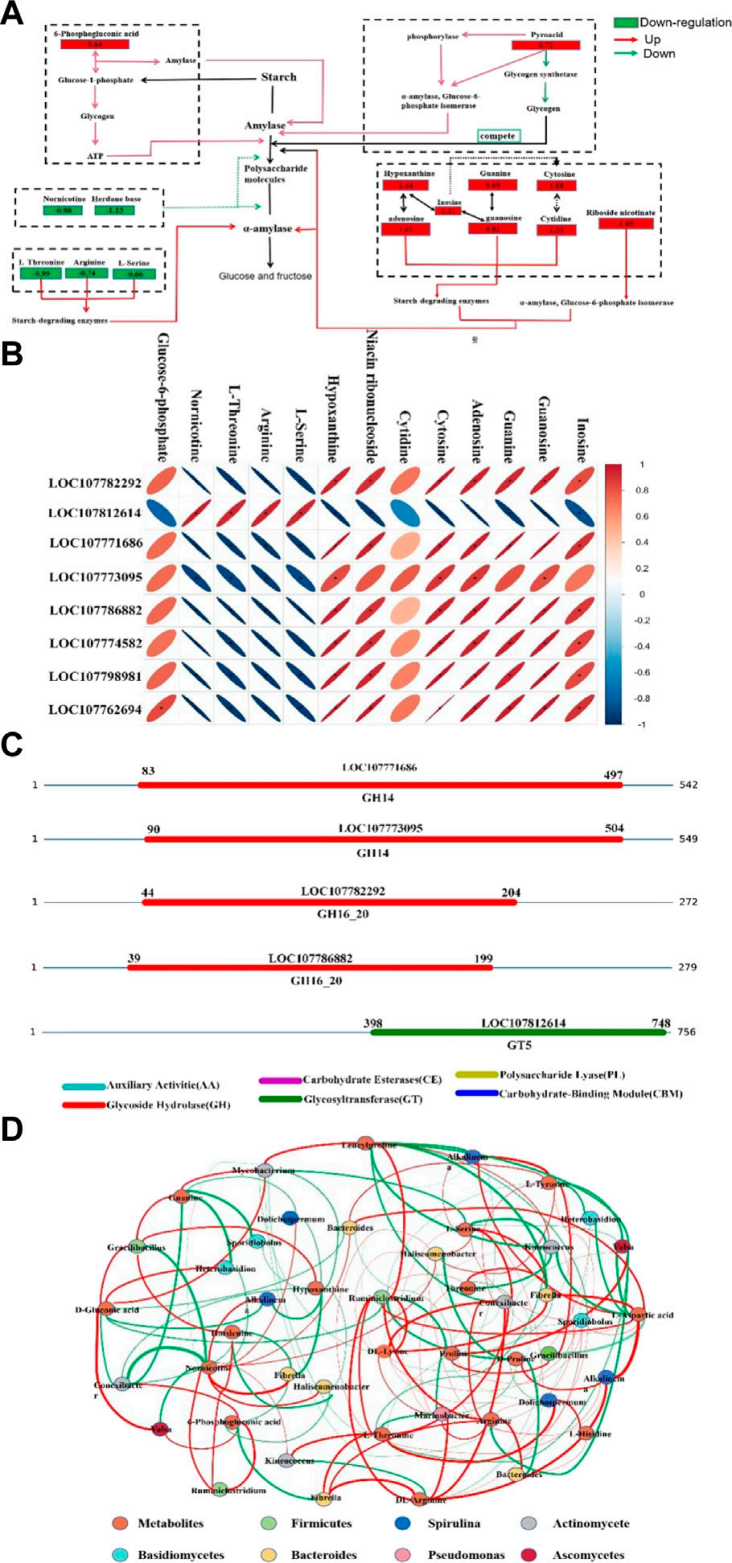
The network analysis **(A)** The network analysis of starch metabolism; The value represents log2FC **(B)** Heat map of association analysis in transcriptome and metabolism. **(C)** the annotation of CAZymes using dbCAN. **(D)** Metagenome and metabolome correlation analysis; Red means positive correlation, Green means negative correlation, The thickness of the line represents the strength of the correlation (*p* < 0.5).

A network related to starch metabolism was constructed (Figure 7A), and the levels of various metabolites within this network were compared. Significant changes in amino acids, alkaloids, organic acids, and nucleotides were observed following the application of BS3. The levels of nornicotine (−0.98), hordanine (−1.13), L-threonine (−0.99), arginine (−0.74), L-serine (−0.66) in the BS3 group were found to be significantly lower than those in the CK group. These amino acids, being important components of starch synthetase, have their metabolism downregulated, which inhibits starch synthesis and indirectly promotes starch degradation (Sehnke et al., 2001). Nornicotine and hordanine, classified as alkaloids, have been reported to inhibit amylase, reducing the rate of starch degradation. The downregulation of these metabolite alkaloids may decrease amylase inhibition, thereby promoting starch degradation (Sampath et al., 2022). In the BS3 group, significant upregulation was observed in the content of glucose-6-phosphate (0.64), pyrogallol (0.71), hypoxanthine (1.44), niacin ribonucleoside (1.65), cytidine (1.35), cytosine (1.68), adenosine (1.01), guanine (0.68), guanosine (0.81), and inosine (0.81). Purine metabolism and pyrimidine metabolism, important in affecting carbohydrates, are known to promote cell energy production and accelerate starch degradation. In conclusion, the application of BS3 is observed to regulate the starch degradation process by altering the starch metabolism network, thereby enhancing the quality of tobacco leaves.

To further elucidate the mechanism of BS3’s role in facilitating starch degradation in tobacco leaves, transcriptomic and metabolomic data were integrated using correlation and network analysis methods (Figure 7B). This analysis revealed significant correlations (*p* < 0.05) between 8 genes and 13 metabolites. Significant negative correlations were observed between key genes (except for *LOC107812614*) and various amino acids (L-threonine, Arginine, L-serine) as well as nicotine. Conversely, significant positive correlations were noted with glucose-6-phosphate and nucleotide metabolites. These genes are closely associated with glycoside hydrolases (*LOC107782292, LOC107771686, LOC107786882,* and *LOC107773095*), starch biosynthesis (*LOC107812614*), and starch plasticity and nucleotide transfer (*LOC107774582, LOC107798981, LOC107762694*).

To further investigate BS3’s promotion of carbohydrate metabolism, a comprehensive annotation of key genes’ CAZymes was conducted using the dbCAN database (Figure 7C), along with an in-depth analysis of transcriptomic data. This revealed 2 CAZyme family members, including 4 genes belonging to the Glycoside Hydrolases (GH) family and 1 to the Glycosyltransferases (GT) family. Significant expression differences were observed in some CAZyme family members in the treatment group, especially for GH14, GH16, and GT5 families, which exhibited a significant increase in expression under BS3 application. Further analysis revealed these CAZymes were closely associated with the degradation of various polysaccharides and oligosaccharides in tobacco.

Changes in metabolites are closely associated with the microbial community. Network association analysis was employed to explore the relationship between microorganisms and the key metabolites mentioned (Figure 6B; Figure 7D). The addition of BS3, in comparison to CK, significantly enhanced the richness and diversity of microorganisms. Microorganisms that strongly interacted with key metabolites were identified as *Firmicutes, Basidiomycetes, Bacteroidetes, actinomycetes,* and *cyanobacteria*. Significant correlations were observed between the changes in amino acid metabolites (L-Aspartic acid, L-Tyrosine, Proline, Threonine, L-Serine, Leucylproline, Arginine, L-Hisidine) in tobacco leaves and the relative abundance of *Gracilibacillus, Conexibacter, Fibrella, Marinobacter, Ruminiclostridium,* and *Sporidiobolus*. The microorganisms were classified as belonging to *Firmicutes, Basidiomycetes, Actinomycetes,* and *Bacteroidetes*. A significant increase was observed in the abundance of *Basidiomycetes* and *Firmicutes*, which was identified as the primary reason for the upregulation of aspartate, leucine, and tryptophan metabolites. The aspartate, leucine and tryptophan metabolites may be used with *Basidiomycetes* and *Bacteroidetes* to synthesize amylase and cellulase (Tanaka et al., 2020; Rangarajan et al., 2022), degrade starch and cellulose, and produce amino acids. A significant decrease was observed in the richness of *Cyanophyta*, which may be the main reason for the reduced expression of arginine, serine, and threonine. As key amino acids in starch synthase, the reduction in cyanobacteria levels leads to decreased starch synthase activity. The metabolism of these amino acids is downregulated, thus reducing starch synthesis (Busi et al., 2014). Significant correlations were observed between the changes in Nornicotinoid and Hordanine alkaloid metabolism and the abundance of G*racilibacillus, Ruminiclostridium, Fibrella, Conexibacter, and Alkalinema. Gracilibacillus,* a genus of *Bacillus*, has been found to inhibit the alkaloid metabolism of microorganisms (Huang S. et al., 2022b). Significant correlations were found between the intermediate products of starch degradation, such as 6-phosphogluconic acid and D-gluconic acid, and the varied abundance of *Conexibacter, Fibrella, Valsa, Ruminiclostridiuma,* which are known to promote the glycolysis and gluconogenesis pathways (Silver et al., 2014). *Valsa*, belonging to the *Ascomycetes*, is known to decompose organic matter, forming glucose-6-phosphate (G6P), and subsequently isomerizing G6P to synthesize enzymes capable of decomposing starch (Sarris and Papanikolaou, 2016).

## 4 Discussion

In this study, a novel functional bacterium, *B. subtilis* strain BS3, was successfully isolated for the first time from the rhizosphere soil of tobacco and the surface of tobacco leaves. This bacterium demonstrated a remarkable ability to degrade starch in tobacco leaves during the curing process. The application of BS3 to tobacco leaves was found to yield promising outcomes, effectively enhancing pertinent substances and enzyme activities, thus significantly accelerating starch degradation. In-depth examinations of the metagenome, transcriptome, and metabolome of tobacco leaves subjected to diverse treatments revealed the mechanism through which BS3 facilitated starch degradation. The composition of dominant bacteria was found to be regulated by the BS3 strain through the modulation of the composition and diversity of foliar microbial communities. For example, the dominance of fungi was observed to shift from basidiomycetes to ascomycetes. This shift promoted the production of enzymes related to starch and cellulose, regulated carbon and nitrogen metabolism, and amino acid and nucleotide metabolism in tobacco leaves. Additionally, it promoted the production of cell energy and accelerated the degradation of starch. Significant changes were observed in the levels of alkaloids, amino acids, and other metabolites, which reduced their influence on the activity of starch-degrading enzymes, subsequently accelerating starch degradation. The isolation, identification, and functional analysis of the BS3 strain have opened new avenues for the extraction of functional bacteria in tobacco, as well as providing valuable insights into their underlying mechanisms. Consequently, this discovery represents a promising strategy for effectively enhancing the quality of tobacco leaves during the curing process.

SOD, POD, MDA, PPO, SBE, α-amylase, β-amylase, and total amylase are important factors affecting starch degradation during tobacco curing (Wang et al., 2021). It was demonstrated in this study that the application of the functional bacterium BS3 suspension to tobacco leaves prior to the curing process significantly affected various enzymatic activities. Enhanced activities of SOD and POD, along with an increase in MDA content, were observed, thereby augmenting the tobacco’s resistance to stress. Simultaneously, a reduction in PPO activity was observed, which enhanced the tobacco’s resistance to roasting and mitigated the occurrence of browning reactions. The activities of α-amylase, β-amylase, and total amylase were found to be enhanced to varying degrees, facilitating the degradation of starch during the curing process. Furthermore, the application of BS3 resulted in an extended period characterized by rapid starch degradation, effectively promoting the breakdown of starch in tobacco leaves.

The composition of the microbial community was found to play a pivotal role in the starch degradation process during tobacco curing. The microbial community was observed to have the ability to regulate the chemical composition and aroma of tobacco leaves, thereby enhancing their overall quality (Zheng et al., 2022). Evidence provided by this study indicated that the application of BS3 during the curing process resulted in substantial changes in the abundance of OTUs on the surface of tobacco leaves, significantly impacting the composition of the microbial community.

In this study, transcriptome analysis was conducted to explore the mechanism behind starch degradation by the functional bacterium BS3. GO enrichment analysis revealed that DEGs were significantly enriched in pathways related to glucosyltransferase activity, polysaccharide metabolism, and glucan metabolism following the application of BS3. n these metabolic processes, genes of the Glycosyl hydrolase family, including *LOC107782292, LOC107786882, LOC107771686,* and *LOC107773095*, were upregulated. Glycosyl hydrolases are responsible for hydrolyzing glycosidic bonds between two or more carbohydrates, or between carbohydrates and non-carbohydrate molecules. An increase in their expression is associated with promoting the degradation of starch (Davies and Henrissat, 1995). In addition, the functions of DEGs were estimated KEGG pathways enrichment analysis. The results indicated that the metabolic pathways of starch and sucrose, amino acid and nucleotide were significantly enriched, and the enrichment of these key pathways was conducive to promoting the degradation of starch into sucrose, glucose and other intermediates (Kafer et al., 2004; Kötting et al., 2010). In the metabolic pathway of Starch and sucrose, the gene of *LOC107812614* related to starch synthase catalytic was downregulated, which could reduce starch synthesis. In amino acid and nucleotide metabolic pathways, In amino acid and nucleotide metabolic pathways, the genes related to the plasticity of starch and the activity of nucleotide transferase (*LOC107774582, LOC107798981, LOC107762694*) were upregulated, which might achieve the degradation of starch by changing the structure of starch and synthesis of nucleotide (Henrissat et al., 1995). These mainly regulate the breakdown of starch in cells.

WGCNA was a progressive analysis method in which variable genes are divided into co-expression modules through an unsigned network based on DEGs (Langfelder and Horvath, 2008). In this study, three key modules were identified that showed significant correlation with the process of starch degradation and the metabolic activities of its related metabolites. Specifically, the purple and tan modules were closely related to the decomposition of organic macromolecules. In these two modules, a series of genes such as *LOC107777055, LOC107796487, LOC107798889* exhibited significant downregulation. These genes are directly associated with the proteasome beta type-1 subunit, suggesting a reduction in starch degrading enzyme activity, which may facilitate effective degradation of starch (Kurepa and Smalle, 2008). Additionally, in the Cyan module, significant enrichment was observed in the pyrimidine and purine metabolic pathways, as well as ATP synthesis pathways. Genes such as *LOC107797877* and *LOC107772372* in this module showed significant upregulation. These genes play a key role in pyruvate metabolism and ATP synthesis, potentially enhancing glycolytic pathways and energy metabolism processes, thereby aiding in the effective breakdown of starch in tobacco (Börnke and Sonnewald, 2011; Santelia et al., 2015).

The annotation of CAZymes using dbCAN is expected to further enhance the understanding of carbohydrate metabolism. It was found that the proteins encoded by the above key genes are primarily glycoside hydrolases and glycosyltransferases. Glycoside hydrolase proteins are responsible for catalyzing the hydrolysis of glycosides, thereby releasing monosaccharides (Minic, 2008). Glycosyltransferases were involved in the synthesis or modification of glycosides. (Wang and Hou, 2009). Following BS3 treatment, a significant upregulation was observed in the expression levels of genes for glycoside hydrolase enzymes, leading to a notable increase in the activity of glycoside hydrolases and glycosyltransferases. This further enhanced the hydrolysis of starch and the metabolic pathways of ribose. Conversely, the expression levels of glycosyltransferase genes were downregulated, reducing the synthesis of large molecules and complex carbohydrates. The genes of *LOC107771686, LOC107773095, LOC107774582* and *LOC107798981* were upregulated in BS3 treatment group. Among them, the genes of *LOC107771686* and *LOC107773095* belong to the Glycoside Hydrolase family 14 (GH14), while *LOC107774582* and *LOC107798981* genes belong to the Glycoside Hydrolase family 16 (GH16) (The CAZypedia Consortium, 2018). Glycoside Hydrolase family is one of the major groups of CAZymes and participate in the catalysis of the glycosidic linkages present between the two monomeric units of the polysaccharides, which played an important role in starch degradation (Herbers et al., 2001). According to reports, GH family-related genes in grapes promote the synthesis of β-amylase, which promotes starch degradation (Zhou et al., 2023). In addition, overexpression of the gene of GH family in rice could accelerate starch degradation efficiency and increase the sugar supply for sink organs (Wang et al., 2022). Therefore, the upregulated expression of *LOC107771686, LOC107773095, LOC107774582* and *LOC107798981* in BS3 treatment group colud accelerate starch degradation. Additionally, this study employed a combined analysis of metabolomic data and transcriptome profiling to identify genes involved in carbohydrate metabolism. Through association analysis, the genes significantly correlated with metabolites were primarily identified as those of the Glycosyl hydrolase family (*LOC107782292, LOC107786882, LOC107771686,* and *LOC107773095*) and starch synthase catalytic (*LOC107812614*). These genes play an important role in regulating carbohydrate metabolism. Glycosyl hydrolases can hydrolyze glycosidic bonds, participating in the degradation of various polysaccharides and oligosaccharides. *LOC107812614,* being one of the key genes of starch synthase, has its downregulation leading to reduced starch synthesis, further promoting starch degradation. Based on the above, it is considered that these genes of the Glycosyl hydrolase family (*LOC107782292, LOC107786882, LOC107771686,* and *LOC107773095*) were the most important in the metabolic process of starch degradation.

Significant changes were found in metabolites involved in starch degradation through metabolome analysis, such as metabolites involved in nucleotide metabolism, which is the substrate for RNA and DNA synthesis and the main source of energy for cellular processes. After BS3 was applied, adenine, cytosine, hypoxanthine and other metabolites were significantly upregulated, accelerating energy production and promoting starch degradation (Kilstrup et al., 2005). Starch degradation intermediates such as G6P are increased. SnRK1 is the target of sugar signaling. G6P can inhibit SnRK1 signaling factor and reduce starch synthesis (Nunes et al., 2013; Silver et al., 2014). It was further proved that the application of BS3 could promote the degradation of tobacco starch. The metabolism level of alkaloids such as Nornicotinine and Hordanine is reduced, and the inhibition of starch degrading enzymes is reduced (Rahman et al., 2019). L-threonine, arginine and L-serine are the key amino acids of starch synthase. The downregulation of these metabolites reduces the synthesis of starch and indirectly promotes the degradation of starch (Sehnke et al., 2001).

## 5 Conclusion

In this study, a suitable functional bacterium, BS3, was isolated and screened from the rhizosphere soil and leaf surface of tobacco, marking the first instance of its discovery. Through identification, BS3 was confirmed to be *B. subtilis*. The application of the BS3 strain on tobacco leaves can regulate the activities of SOD, POD, PPO, SBE, α-amylase, β-amylase, total amylase, and the content of MDA, thereby enhancing the degradation of starch in tobacco leaves. Moreover, the application of BS3 contributes to the amelioration of the tobacco leaf metagenome composition and facilitates the regulation of starch metabolism pathways, thereby facilitating the process of starch degradation. This study screened successfully a functional bacterium which can degrade starch efficiently. The mechanism of starch degradation by the functional bacterium was successfully analyzed, which provided a novel direction for the extraction of functional bacteria from tobacco leaves and the elucidation of their mechanisms of action. It offered new strategies for efficiently improving the quality of tobacco leaves.

## Data Availability

The data presented in the study are deposited in the NCBI repository, the accession number of 16S and RNA-seq was OR939273 and PRJNA1053396.
