# Supplementary material for: Screening, identification, and mechanism analysis of starch-degrading bacteria during curing process in tobacco leaf
Source: Front Bioeng Biotechnol. 2024 Mar 19;12:1332113. doi: 10.3389/fbioe.2024.1332113 (PMC10985783; doi:10.3389/fbioe.2024.1332113)
Supplement: Supplementary file 1 [file Table1.DOCX]

Table S1 Starch degradation strains are screened

| Serial number | the diameter of the single colony degradation zone  H（mm） | Single colony diameter  C（mm） | H/C | Enzyme activity（U/mL） |
| --- | --- | --- | --- | --- |
| 1 | 9.83 | 5.65 | 1.75 | 72.96 |
| 2 | 5.84 | 3.64 | 1.65 | 106.71 |
| 3 | 10.18 | 7.16 | 1.43 | 89.84 |
| 4 | 7.54 | 3.86 | 1.98 | 62.84 |
| 5 | 4.18 | 2.24 | 1.88 | 62.84 |
| 6 | 9.97 | 6.75 | 1.48 | 96.59 |
| 7 | 4.47 | 2.32 | 1.96 | 79.71 |
| 8 | 3.88 | 3.4 | 1.14 | 86.84 |
| 9 | 4.47 | 2.32 | 1.96 | 86.84 |
| 10 | 16.39 | 9.35 | 1.78 | 103.34 |
| 11 | 12.84 | 7.27 | 1.8 | 79.71 |
| 12 | 7.64 | 2.56 | 3.04 | 150.59 |
| 13 | 7.9 | 3.18 | 2.6 | 116.84 |
| 14 | 3.84 | 1.42 | 2.74 | 126.96 |
| 15 | 7.96 | 6.28 | 1.26 | 62.84 |
| 16 | 4.54 | 2.82 | 1.61 | 62.84 |
| 17 | 9.8 | 4.49 | 1.52 | 72.96 |
| 18 | 10.36 | 6.14 | 1.73 | 89.84 |
| 19 | 10.82 | 9.96 | 1.09 | 62.84 |

Table S2 The degradation rate of tobacco leaf starch at different times

| Time | CK | T1 | T1 Degradation rate | T3 | T3 Degradation rate |
| --- | --- | --- | --- | --- | --- |
| 2h | 101.6a | 93.71a | 7.71% | 100.36a | 1.21% |
| 12h | 97.05a | 86.53a | 10.84% | 89.43a | 7.85% |
| 24h | 94.44a | 83.37ab | 11.72% | 85.03b | 9.97% |
| 48h | 92.82a | 77.1b | 16.93% | 68.83b | 25.8% |
| 72h | 87.66a | 68.12b | 22.29% | 63.44b | 27.64% |


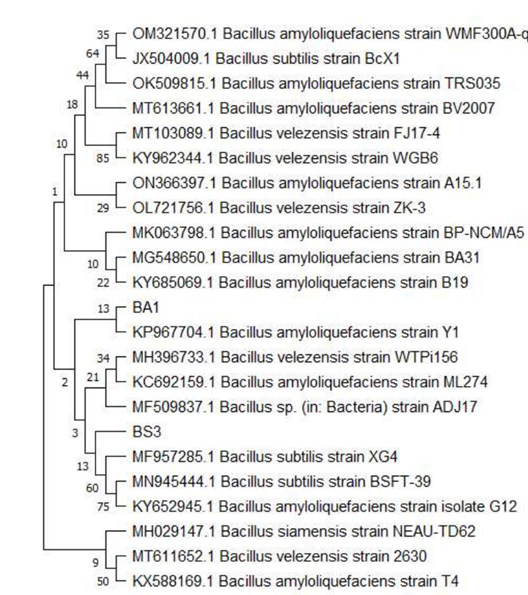


Figure S1 Phylogenetic tree based on 16S rDNA sequences

Table S3 α diversity index of bacteria

| Constituencies | Chao1 | OTUs | Shannon | Simpson | Coverage |
| --- | --- | --- | --- | --- | --- |
| CK | 203.78±46.49 | 199±42.57 | 3.49±0.73 | 0.93±0.00* | 0.9997 |
| BS3 | 263.58±41.34 | 259±40.73 | 5.18±0.31* | 0.72±0.11 | 0.9996 |

Note: ** indicates a very significant difference (P<0.01), * indicates a significant difference (P<0.05).

Table S4 α diversity index of fungi

| Constituencies | Chao1 | OTUs | Shannon | Simpson | Coverage |
| --- | --- | --- | --- | --- | --- |
| CK | 156.52±49.34* | 153.33±46.5* | 2.4±1.77 | 0.84±0.00 | 0.9998 |
| BS3 | 64.66±21.1 | 63.66±22.36 | 3.73±0.22 | 0.58±0.44 | 0.9999 |

Note: ** indicates a very significant difference (P<0.01), * indicates a significant difference (P<0.05).
